# Editorial: Endocrine malignancies: from pathophysiology to current clinical and surgical therapeutic approaches

**DOI:** 10.3389/fonc.2023.1220372

**Published:** 2023-06-20

**Authors:** M. Tarallo, L. Petramala, B. Altieri

**Affiliations:** ^1^ Department of Surgery, Sapienza University of Rome, Rome, Italy; ^2^ Department of Translational and Precision Medicine, Sapienza University of Rome, Rome, Italy; ^3^ Division of Endocrinology and Diabetology, Department of Internal Medicine I, University Hospital of Wuerzburg, Würzburg, Germany

**Keywords:** endocrine tumor, thyroid, parathyroid, adrenal gland, endocrine surgery

Endocrine tumors of the thyroid, parathyroid, and adrenal glands are a significant and complex medical issue that affects millions of people worldwide. These tumors can lead to a variety of serious conditions, including hyperthyroidism, hypothyroidism, hyperparathyroidism (1), and pheochromocytoma (2); moreover endocrine tumors can be benign or malignant, and their diagnosis and treatment require a multidisciplinary approach involving endocrinologists, radiologists (3), pathologists, and surgeons. In recent years, significant progress has been made in our understanding of the pathophysiology of endocrine tumors, as well as in the development of new diagnostic and therapeutic approaches. Hence, this Research Topic aims to provide an up-to-date overview of endocrine tumors, from their pathophysiology to current clinical and surgical therapeutic approaches. The Research Topic includes contributions from leading experts in the field, covering a broad range of topics related to endocrine tumors. The articles in this Research Topic cover the variability of development and the importance of the latest advances in managing thyroid tumors and its debated topics such as lymph node metastasis, central neck dissection, micrometastasis and ectopic tumors.

From the application of the 2015 American Thyroid Association (ATA) guidelines, Wu et al. have retrospectively analyzed a large cohort of differentiated thyroid cancer patients from Wuhan Union Hospital (WHUH), finding that among all factors, age <35 years, clinical N1, and ultrasound reported local invasion had high positive predictive value to predict patients who should undergo total thyroidectomy; as regard Authors have suggested two new models of management, evaluating nodule size (cut off 4 cm), age (cut off 35 years old) in order to achieve better sensibility and sensitivity.

With the aim to develop the nomograms for Lateral Lymph Node Metastasis (LLNM) and to determine predictive factors in patients with papillary thyroid carcinoma and microcarcinoma, Feng et al. reviewed the medical records of a large cohort of patients. Authors suggest treatment protocols for postoperative management of PTC patients with different risks.

The lymph node metastases of papillary thyroid cancer represent another important topic since they can have different locations and the management of these patients and their follow-up must have particular attention.

Wang et al. conducted a metanalysis to study the relationship between pretracheal and/or prelaryngeal lymph node metastasis and paratracheal and lateral lymph node metastasis; while a singular clinical case of papillary thyroid microcarcinoma patient with skip lymph node metastasis (lateral cervical lymph node metastasis without central lymph node metastasis) and multiple distant metastasis in lung and bone has reported by Jiang et al.

Papillary thyroid cancer is a disease that can also affect children. Ngo et al. has evaluated Pediatric Papillary Thyroid Cancer, rare condition, especially regarding safety of prophylactic central neck dissection (CND) respect of disease-free survival (DFS), pointing out that this procedure is associated with increased DFS and not with increased rates of complications after surgery.

Thyroid cancer can be ectopic although rare (0.3%–0.5% of thyroid cancer). Fu et al. performed a literature review reporting 132 clinical cases of Ectopic Thyroid Cancer (ECT), adding the personal case report of 13-years-old girl with thyroid cancer of the deep surface of the tongue base. Authors stated that surgery with complete resection is the main treatment for ETC.

The Research Topic also includes an interesting perspective of current surgical innovations for thyroid cancer management, highlighting the latest surgical techniques and strategies.

Zhang et al. has researched further studies to examine the effect of selective inferior parathyroid gland autotransplantation on central lymph node dissection (CLND) and incidence of postoperative hypoparathyroidism in patients undergoing endoscopic radical resection of thyroid carcinoma. These Authors have suggested that in patients undergoing endoscopic radical resection of thyroid carcinoma, the parathyroid autotransplantation is more beneficial to postoperative parathyroid glands function recovery, effectively preventing postoperative permanent hypoparathyroidism.

Princi et al. critically examined the experience of the intraoperative nerve monitoring (IONM), an innovative surgical instrument, in a referral center for thyroid and parathyroid surgery. Princi et al. reported surgical outcomes comparing two groups of patients (IONM group and control group). They reported no differences in terms of temporary or definitive recurrent laryngeal nerve injury and stated that routine use of IOMN increases the surgery cost, but overall, it leads to the reduction of both operating times and length of Hospital stay.

Other topics covered in this Research Topic include the treatment of parathyroid tumors. Alberti et al. performed a systematic review of published cases of metastatic parathyroid carcinoma. It has a poor prognosis and the main goals of treatment are to neutralize tumor growth and control hypercalcemia; nevertheless uncontrolled hyperparathyroidism remain the main cause of death. The study emphasized surgery of metastases as the best approach, with a better OS; target therapies and immunotherapy deserve to be extensively tested.

At last, but not least, the Research Topic includes some valuable experiences in the treatment of adrenal tumors and the importance of multidisciplinary care in managing these complex conditions.

Luo et al. described two cases: 50-year-old which developed acute respiratory distress syndrome (ARDS) requiring mechanical ventilation after pheochromocytoma rupture; 46-year-old woman admitted in hospital for pulmonary edema after intrauterine device removal (by hysteroscopy) and occasional find of pheochromocytoma; in both cases laparoscopic adrenalectomy was associated to success after adequate preoperative medical management.

Dong et al. have evaluated 5 cases and analyzed the literature on the management of oncocytic adrenocortical neoplasms, rare and mostly benign tumors, underlining that the surgical resection is the main treatment method, but a careful pathological examination and close follow-up are needed to confirm the prognosis.

Overall, this Research Topic has provided a valuable resource for clinicians and researchers working in the field of endocrine tumors. We hope that readers appreciated this Research Topic “*Endocrine tumors: from pathophysiology to current clinical and surgical therapeutic approaches*.” By bringing together the latest research and clinical knowledge, we hope we have advanced our understanding of these complex neoplasms and improved outcomes for patients with endocrine tumors.

## Author contributions

Article writing: MT and LP; draft manuscript preparation: MT. All authors contributed to the article and approved the submitted version.
